# High-Density Blood Transcriptomics Reveals Precision Immune Signatures of SARS-CoV-2 Infection in Hospitalized Individuals

**DOI:** 10.3389/fimmu.2021.694243

**Published:** 2021-07-16

**Authors:** Jeremy W. Prokop, Nicholas L. Hartog, Dave Chesla, William Faber, Chanise P. Love, Rachid Karam, Nelly Abualkheir, Benjamin Feldmann, Li Teng, Tamara McBride, Mara L. Leimanis, B. Keith English, Amanda Holsworth, Austin Frisch, Jacob Bauss, Nathisha Kalpage, Aram Derbedrossian, Ryan M. Pinti, Nicole Hale, Joshua Mills, Alexandra Eby, Elizabeth A. VanSickle, Spencer C. Pageau, Rama Shankar, Bin Chen, Joseph A. Carcillo, Dominic Sanfilippo, Rosemary Olivero, Caleb P. Bupp, Surender Rajasekaran

**Affiliations:** ^1^ Department of Pediatrics and Human Development, College of Human Medicine, Michigan State University, Grand Rapids, MI, United States; ^2^ Department of Pharmacology and Toxicology, Michigan State University, East Lansing, MI, United States; ^3^ Allergy & Immunology, Spectrum Health, Grand Rapids, MI, United States; ^4^ Office of Research, Spectrum Health, Grand Rapids, MI, United States; ^5^ Department of Obstetrics, Gynecology and Reproductive Biology, College of Human Medicine, Michigan State University, Grand Rapids, MI, United States; ^6^ Physical Sciences, Grand Rapids Community College, Grand Rapids, MI, United States; ^7^ Ambry Genetics, Aliso Viejo, CA, United States; ^8^ Pediatric Intensive Care Unit, Helen DeVos Children’s Hospital, Grand Rapids, MI, United States; ^9^ The Department of Chemistry and Biochemistry, Calvin University, Grand Rapids, MI, United States; ^10^ Department of Biology, Grand Valley State University, Allendale, MI, United States; ^11^ Department of Science, Davenport University, Grand Rapids, MI, United States; ^12^ Department of Critical Care Medicine and Pediatrics, Children’s Hospital of Pittsburgh, University of Pittsburgh School of Medicine, Pittsburgh, PA, United States; ^13^ Infectious Disease, Helen DeVos Children’s Hospital, Grand Rapids, MI, United States; ^14^ Medical Genetics, Spectrum Health Medical Genetics, Grand Rapids, MI, United States

**Keywords:** COVID-19, SARS-CoV-2, blood transcriptomics, RNAseq, immune repertoire, interferon response, immune cell deconvolution, secondary infections

## Abstract

The immune response to COVID-19 infection is variable. How COVID-19 influences clinical outcomes in hospitalized patients needs to be understood through readily obtainable biological materials, such as blood. We hypothesized that a high-density analysis of host (and pathogen) blood RNA in hospitalized patients with SARS-CoV-2 would provide mechanistic insights into the heterogeneity of response amongst COVID-19 patients when combined with advanced multidimensional bioinformatics for RNA. We enrolled 36 hospitalized COVID-19 patients (11 died) and 15 controls, collecting 74 blood PAXgene RNA tubes at multiple timepoints, one early and in 23 patients after treatment with various therapies. Total RNAseq was performed at high-density, with >160 million paired-end, 150 base pair reads per sample, representing the most sequenced bases per sample for any publicly deposited blood PAXgene tube study. There are 770 genes significantly altered in the blood of COVID-19 patients associated with antiviral defense, mitotic cell cycle, type I interferon signaling, and severe viral infections. Immune genes activated include those associated with neutrophil mechanisms, secretory granules, and neutrophil extracellular traps (NETs), along with decreased gene expression in lymphocytes and clonal expansion of the acquired immune response. Therapies such as convalescent serum and dexamethasone reduced many of the blood expression signatures of COVID-19. Severely ill or deceased patients are marked by various secondary infections, unique gene patterns, dysregulated innate response, and peripheral organ damage not otherwise found in the cohort. High-density transcriptomic data offers shared gene expression signatures, providing unique insights into the immune system and individualized signatures of patients that could be used to understand the patient’s clinical condition. Whole blood transcriptomics provides patient-level insights for immune activation, immune repertoire, and secondary infections that can further guide precision treatment.

## Introduction

Coronavirus Disease of 2019 (COVID-19), caused by the ~30kb single-stranded RNA virus known as SARS-CoV-2, primarily infects the respiratory system resulting in a variety of pulmonary insults ranging from pneumonitis to acute respiratory distress syndrome (ARDS) (1). COVID-19 involves responses from both innate and adaptive immune system branches, leading both to an overactive inflammatory process and clonal lymphocyte anti-SARS-CoV-2 antibodies (2). Patients with higher severity of illness have elevated blood pro-inflammatory cytokines, secondary hemophagocytic lymphohistiocytosis, hyperferritinemia, and cytokine storm, resulting in end-organ damage (3–5). It is not well understood why some individuals are asymptomatic, yet others advance into multiorgan failure and die. Upon entering the respiratory epithelial cells through the ACE2 receptor, the virus can evade intracellular detection (6), causing an altered type I interferon (IFN) response (7). The adverse immune response is characterized by an increased risk of secondary infections, lymphopenia, over-activation of neutrophil mechanisms, and increased systemic coagulation issues leading to end-organ injury (8–10). Several immune system components have been identified to have genetic variants that contribute to severity (11, 12).

Yet, despite these observations and mechanistic understanding, the range of clinical variability in affected patients is vast. This unpredictability of disease course, comorbidities, and severity create challenges for understanding individual vulnerability and determining when and if appropriate therapies need to be initiated (13). In choosing the right treatment strategy for the immune-mediated complications of coronavirus infection, neutralizing the “cytokine storm” is often the putative goal. Still, it is vital to recognize immune incompetence that turns into immuno-paralysis with increased vulnerability to secondary infections (14). In addition to acute complications, the global activation of the immune system may be related to poorly understood chronic problems in survivors of COVID-19 (15, 16) that are beginning to surface in COVID-19 “long-haulers.” The altered immune responses, if identified early, could provide therapeutic options. This has highlighted the further need for developing and testing precision medicine-based tools to understand pathology in each patient using easier to obtain biomaterials such as blood.

Immunomodulatory strategies appear to be promising in patients with severe COVID-19 disease (17). Many different immunomodulatory strategies have been studied, but this requires early identification of the specific patient subgroups who could benefit from their use. The transcriptome has the utility of understanding immune system interactions with various viral infections (18), especially if these tools can be applied to biomaterials such as blood collected into sample tubes that are easy to process, such as the PAXgene tube. Here, we present the highest density (most sequenced bases per patient) blood PAXgene tube transcriptome analysis of severely ill patients hospitalized with COVID-19. We explore the ability to detect multiple insights from the sequenced RNA bases. The work highlights the diverse mechanisms of systemic immune activation and repression while uncovering the heterogeneity of multiple data analyses, all of which suggest the need for further development of precision transcriptomic tools for COVID-19 patients and the immune response. It provides proof that blood-based PAXgene tube RNAseq combined with advanced bioinformatics can give precision medicine insights into each patient impacted by an infectious agent in a way not before appreciated.

## Methods

Patients were consented and samples collected as approved by the Spectrum Health IRB. Inclusion criteria included a PCR positive SARS-CoV-2 clinical test, above 18 years of age, hospitalized with COVID-19. Exclusion criteria included patients with neoplasms, autoimmune disease, immunosuppressive or immunodeficient state, human immunodeficiency virus (HIV) infection, asplenia, recurrent severe infections, or systemic immunosuppressants or immune-modifying drugs for >14 days in total within six months before enrolling. Patients that tested SARS-CoV-2 positive by PCR test had 2mL of blood drawn into PAXgene™ Blood RNA tubes (PreAnalytiX). Control samples received a negative SARS-CoV-2 PCR test and showed no signs of lung pathologies. RNA was isolated from tubes using Thermo MagMAX™ Isolation Kit on Thermo KingFisher Presto. RNA yield was quantified using M200 Infinite^®^ (Tecan) and sequencing libraries prepared using a Globin-minus, RiboErase, RNA HyperPrep Kit (KAPA Biosystems) as previously described (19). Whole transcriptome sequencing was done using 150 bp, paired-end reads on a single Illumina NovaSeq6000 sequencing run on a 300-cycle S4 flow cell to remove batch effect.

A total of 51 patients were consented, blood collected, and RNAseq performed for 74 transcriptomes, where some patients had multiple time points of collection (1 patient with 4 collections, 3 with 3, 14 with 2, 33 with 1). Of these patients, 15 were suspected COVID-19 negative (asymptomatic controls), and 36 were COVID-19 positive as determined by clinical PCR test. Of the 36 COVID-19 positive patients, 11 had mortality. Patients were 21-95 years of age, with 30/51 male and 21/51 female (**Table 1**). The mean age of 61 for the cohort shows the focus on sample collection for hospitalized non-elderly individuals. Moreover, 13/36 COVID-19 positive patients required mechanical ventilation. Simplified Acute Physiology Score (SAPSII) was collected to estimate probability of mortality amongst the patients. Admissions diagnoses included COVID-19 (47%), hypoxia (22%), pneumonia (19%), ARDS (17%), and hypertension (14%). A total of 42% of the COVID-19 patients had heart disease as a pre-existing condition, with lung disease at 25%, hypertension at 19%, and diabetes at 19%.

**Table 1:** Patient demographics of COVID-19 Cohort 2020 (N=51).

	Range	(n)	%	mean	median	st.dev.
**Age** (Years)	21-95	51	100	55	56	18
Controls	21-59	15		40	41	11
COVID+	24-95	36		61	62	17
**Sex**						
Male		30	58.82			
Controls		5				
COVID+		25				
Female		21	41.18			
Controls		10				
COVID+		11				
**Race**						
Controls		15				
Caucasian		5	33.30			
Black		2	13.33			
Hispanic		7	46.67			
Multiracial		0	0			
Other		1	6.67			
COVID+		36				
Caucasian		21	58.33			
Black		7	19.44			
Hispanic		6	16.67			
Multiracial		1	2.78			
Other		1	2.78			
**BMI (**kg/m^2^ **)**	16.11-45.14	51		30.68	28.48	9.12
Controls	20.74-38.52	15		28.63	28.02	4.89
COVID+	16.11-45.14	36		31.54	28.74	0.32
SAPS II Score*						
COVID+	27-66	36		40.00	33.00	12.92
**Ventilation^a^**						
Controls		15				
Mechanical		0	0			
Room Air		15	100			
COVID+		36				
Mechanical		13	36.11			
Non-Invasive		23	63.89			
**Hospital LOS (days)**						
Controls	0	15		0	0	0
COVID+	2-40	36		14.97	13.50	9.79
**Mortality^b^**						
Controls		0	0			
COVID+		11	30.56			

*Simplified Acute Physiology Score; this score estimates the probability of mortality with ICU patients. SAPSII score was not calculated for Control patients ^a^Patients on Room Air did not receive oxygen therapy during stay. ^b^(n) represents total group number.

Paired-end fastq reads were quasi-aligned to *Homo sapiens* Gencode v35/38 or our single-gene to sequence transcriptome (15) using salmon_0.14.1 (20). Mapped transcripts per million (TPM) for all samples were processed through NetworkAnalyst3.0 (21) using Limma (22) to identify gene-level annotations that differ between groups. Pathways identified in NetworkAnalyst to have variable gene expressions with an adjusted p-value (< 0.01) and log2 fold change of 2 were extracted for Gene Ontology (GO) enrichment. Sex of each sample was determined by the presence of multiple chromosome Y genes within RNAseq. CIBERSORTx digital cytometry cell fraction imputation (23) was performed using gene mapping on LM22 signature matrix without batch corrections and 500 permutations.

Bacterial and viral read mapping was performed using kraken2-2.0.7-beta (24) using PlusPFP database, with mapping normalized to every million mapped human reads. The immune repertoire was mapped in each sample using the MiXCR tools (25). Cell-type-specific genes were identified from the human protein atlas (HPA) (26) single cell type atlas. The tissue or blood tools of HPA were used to address significant genes in our samples to determine cell type origins. From the gene level annotation, z-scores were calculated for each gene TPM value across all samples ([Patient gene-level - Gene average]/standard deviation of gene values). Genes for each patient greater than 1 TPM and four standard deviations were identified and assessed with STRING (27) for pathway enrichment. The top 1,000 SNPs from the COVID-19 Host Genetics Initiative (HGI) (12) were extracted from the A2_ALL very severe respiratory confirmed COVID *vs.* population dataset, including 23andMe. The data was processed through SNPnexus (28) to list possible connected genes, followed by analysis with our RNAseq.

## Results

### Transcriptome Statistics

**Figure 1:**
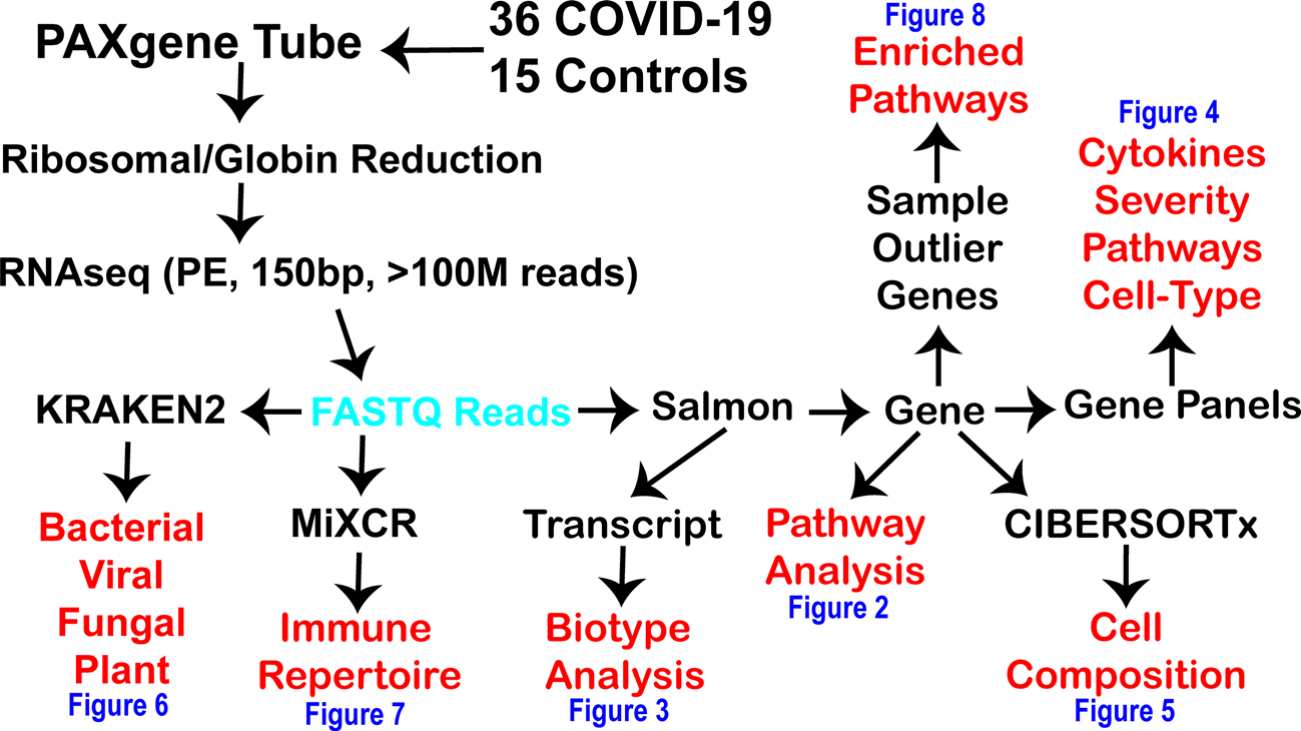
Workflow for precision high-density transcriptomics used within the current study. The black text is the various steps used within the current study to generate and process fastq reads (cyan) for multiple levels of insights (red) shown within the various figures of the paper (blue). RNAseq was done in paired-end (PE) with 150 basepair (bp) reads with at least one hundred million reads generated per patient.

**Figure 2:**
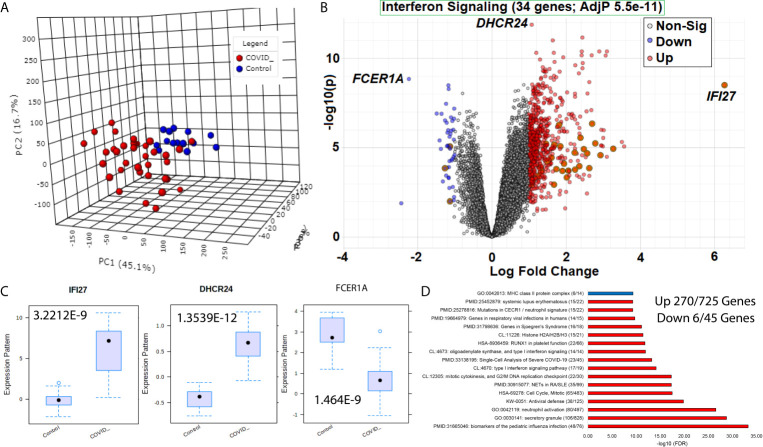
Blood transcriptome gene signatures of SARS-CoV-2 patients. **(A)** Three dimensions of principal components (PC1-PC3) of first collection time point RNAseq gene annotations for samples of COVID-19 (red) or control (blue) patients. **(B)** Volcano plot of gene expression in COVID-19 or control patients with significant genes higher (red) or lower (blue) marked. The x-axis shows the log2 fold change, and the y-axis shows the -log10 of the adjusted p-value. Shadowed genes are involved in interferon signaling. **(C)** Box and whisker plot of the top three genes labeled in panel **(B, D)** Top gene ontology (GO) enrichment terms for up (red) or down (blue) genes. The term for each enriched description is shown first, followed by the name, and then in parentheses, the number of genes is significant relative to the number of genes within the genome annotated for the term. The x-axis shows the -log10 of the false discovery rate (FDR).

**Figure 3:**
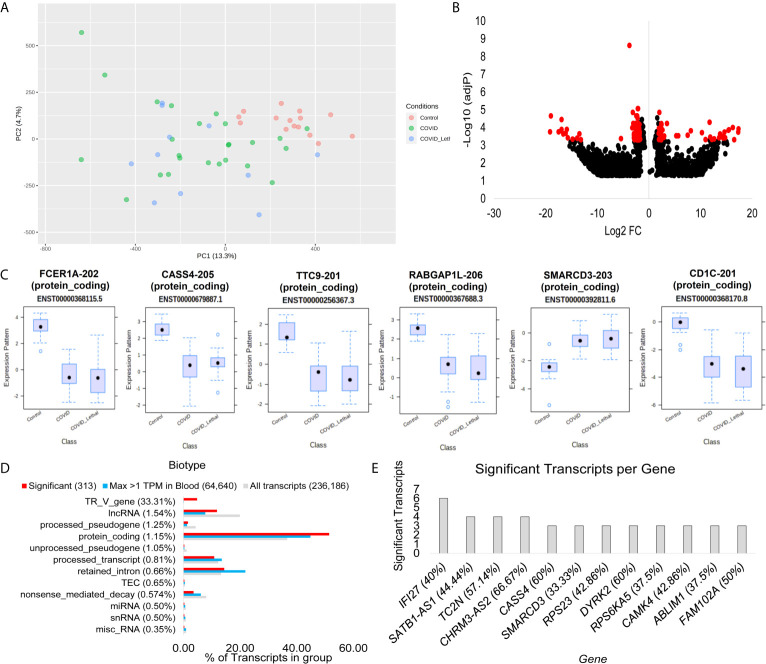
Blood transcriptome transcript isoform signatures of SARS-CoV-2 patients. **(A)** Two dimensions of principal components (PC1-PC2) of first collection time point RNAseq transcript annotations for samples of control (red), COVID-19 and survived (green), or COVID-19 and lethal (blue). **(B)** Volcano plot of transcript expression in COVID-19 or control patients with significant genes with a Log2 of 2 and adjP <0.0005 marked in red. The x-axis shows the log2 fold change, and the y-axis shows the -log10 of the adjusted p-value. **(C)** Box and whisker plot of the top six transcripts, with each having the transcript identifier, biotype, and Ensembl transcript ID listed. **(D)** Top biotypes enriched in the significant transcripts for COVID-19, with the percent listed in paratheses for each biotype annotation. The top biotypes are listed based on enrichment of significance annotation (red) relative to detection within blood within at least one sample with >1TPM (cyan). The percent of transcripts for the entire Gencode 38 database of 236,186 known transcripts is shown in gray. **(E)** The number of transcripts significantly different in the COVID-19 group relative to controls for each gene. The percent of transcripts identified significant relative to all known transcripts for each gene is shown in paratheses.

**Figure 4:**
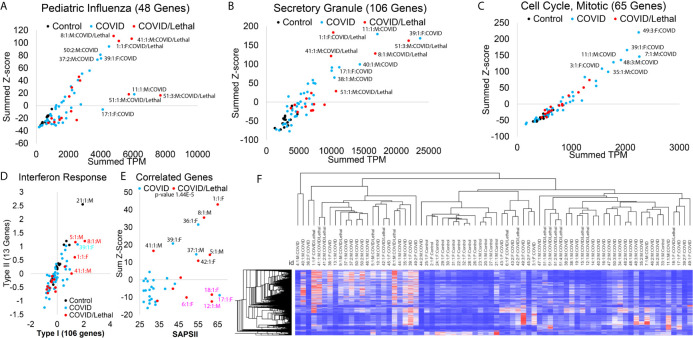
Blood transcriptome gene panel signatures of SARS-CoV-2 patients. **(A–C)** Enriched associated genes for severe pediatric influenza **(A)**, secretory granule **(B)**, and mitotic cell cycle **(C)**. The x-axis shows the added transcript per million (TPM) of all genes in the gene list, and the y-axis shows the added z-score for each group’s genes. Control samples are shown in black, COVID-19 patients in cyan, and COVID-19 patients deceased in red. The outlier samples are labeled (Sample #:collection #:Sex : Group). **(D)** Average Z-score for genes specifically activated to Type I IFN (x-axis) or Type II IFN (y-axis). **(E)** The analysis of SAPSII (Simplified Acute Physiology) relative to combinations of genes that are correlated 0.5-0.4 to the SAPSII. **(F)** Heat map of the 770 significant genes in all samples. Clustering dendrogram of rows and columns is based on Spearman’s rank correlation.

**Figure 5:**
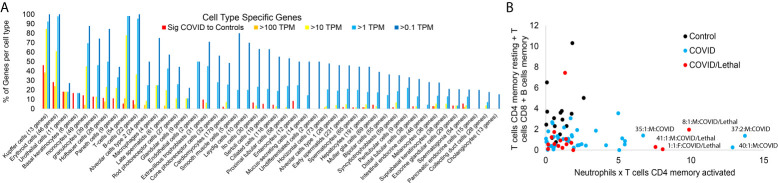
Blood transcriptome cell-specific gene signatures of SARS-CoV-2 patients. **(A)** The number of genes unique to various cell types (x-axis) expressed in the significant group of **Figure 1** (red) or have expression in one or more of the samples greater than 100 TPM (orange), >10 TPM (yellow), or >1 TPM (cyan). **(B)** Absolute values of CIBERSORTx additive for resting CD4 T-cells, CD8 T-cells, and memory B-cells relative to neutrophil and CD4 memory T-cells within the control (black), COVID-19 (cyan), or COVID-19 lethal (red).

**Figure 6:**
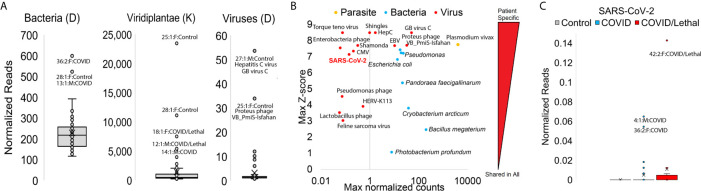
Blood transcriptome foreign RNA signatures of SARS-CoV-2 patients. **(A)** Box and whisker plots for normalized reads mapped to bacteria, plants (Viridiplantae), or viruses with outlier samples labeled. **(B)** Top mapping values in samples for parasite (orange), bacteria (cyan), or virus (red) RNA. Values are shown as the highest (max) sample normalized counts (x-axis) for each labeled *vs.* the z-score for that sample (y-axis) relative to the entire cohort. **(C)** Box and whisker plot for normalized SARS-CoV-2 reads. Controls are gray, COVID samples cyan, and COVID/Lethal red.

**Figure 7:**
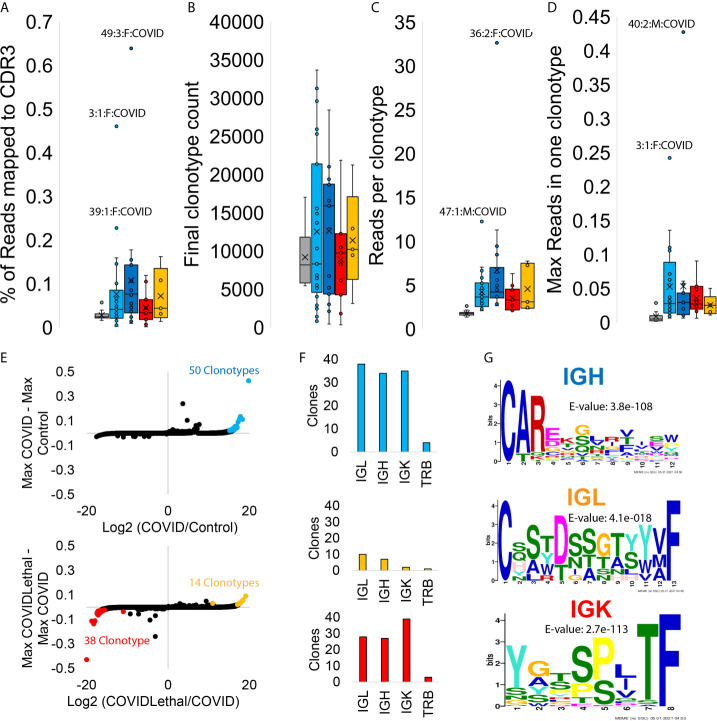
Blood transcriptome immune repertoire signatures of SARS-CoV-2 patients. **(A–D)** Box and whisker plots for statistics for MiXCR CDR3 analysis. Samples are grouped as controls (gray), COVID first collection (cyan), COVID later collections (blue), COVID/Lethal first collection (red), or COVID/Lethal later collections (orange). Data is shown for the % of reads mapped to CDR3 from all reads **(A)**, the number of clonotypes per sample **(B)**, the number of reads per clonotype per patient **(C)**, and the max number of reads within one of the clonotypes for each patient **(D)**. **(E)** The log2 fold change (x-axis) of group averages relative to the max values (y-axis) for each clonotype in all COVID samples *vs.* Controls (top) or Lethal COVID *vs.* non-Lethal COVID (bottom). Outlier clonotypes enriched in one of the groups are labeled (cyan, red, orange). **(F)** Clones labeled in panel **E** (cyan, red, orange) analyzed through CDR3 types (IGL, IGH, IGK, TRB). **(G)** Meme motifs for labeled CDR3 types of extracted clones. Color of IGH/IGL/IGK corresponds to the clones extracted from the colored outliers of panel **E**.

**Figure 8:**
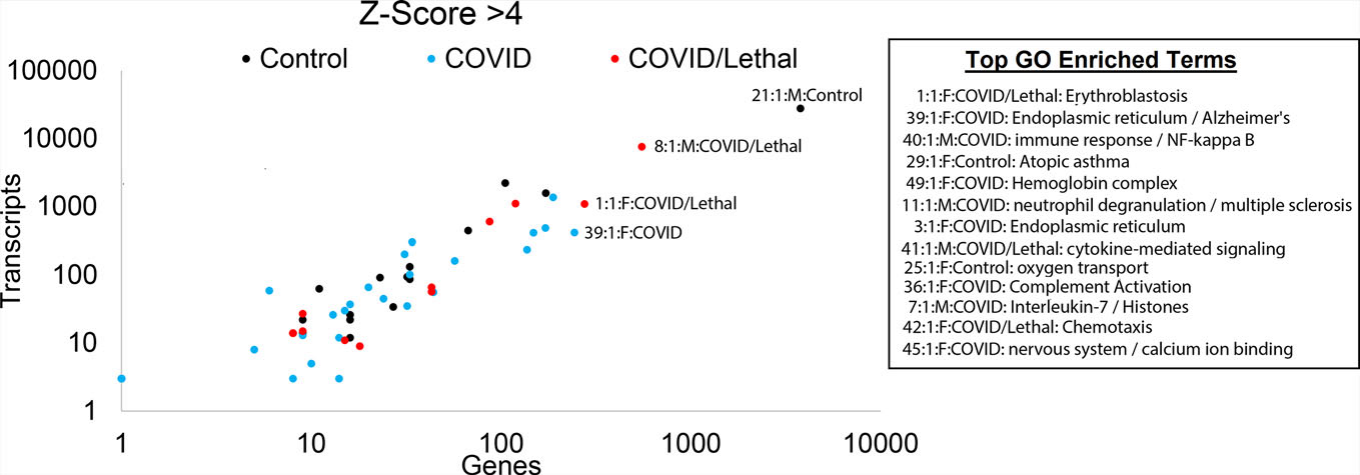
Blood transcriptome outlier gene/transcript signatures of SARS-CoV-2 patients. The number of genes (x-axis) and transcripts (y-axis) in each sample that has a z-score >4. Those samples with enriched gene ontology terms are shown.

The RNAseq sequenced 11,927,375,976 clusters for an average of 161.18 ± 21.49 million clusters per sample. We averaged 97.02% of the sequences with perfect matching barcodes and a mean quality score of 35.59 ± 0.07, meaning the sequencing was performed to a high level with low error. This represents a high-density and quality dataset of COVID-19 and control whole-blood total RNAseq. In comparison, 76 BioProject have been completed and made publicly available in NCBI SRA for blood PAXgene tube RNAseq in paired-end (**Table 2**). This current study generated an average of 45 billion bases of sequencing for each patient, more than 10 billion higher than any other study performed to date. Having this valuable resource with extreme coverage, one can imagine creating a program focused on exploring all the origins of RNA within the blood that can be sequenced, gaining valuable insights into biomarkers and precision medicine. With this depth of sequencing within each patient, we sought out to define the insights that one can gain using advanced bioinformatics (**Figure 1**) for genes (**Figure 2**), transcripts (**Figure 3**), gene panels (**Figure 4**), cell composition (**Figure 5**), foreign RNA including secondary infections (**Figure 6**), the immune repertoire (**Figure 7**), and patient-specific biology (**Figure 8**) applied to hospitalized COVID-19 patients.

**Table 2:** Public datasets found in each BioProject on the NCBI SRA (as of June 2021) for blood PAXgene tube RNA done in paired end with fastq files available.

BioProject	# Blood Samples	Study	Total spots	Total bases	Bases per Sample
PRJNA691933	74	SARS-CoV-2 (Current Study)	11,927,375,976	3,336,510,420,209	45,087,978,651
PRJNA588242	100	tuberculous	17,441,858,528	3,473,056,592,305	34,730,565,923
PRJNA358580	16	pregnant women	1,507,551,371	304,525,376,942	19,032,836,059
PRJNA521868	6	multiple sclerosis	466,613,597	93,322,719,400	15,553,786,567
PRJNA702558	95	SARS-CoV-2	4,774,111,508	1,441,781,675,416	15,176,649,215
PRJNA647880	105	sepsis	5,035,786,125	1,510,735,837,500	14,387,960,357
PRJNA341405	44	Malaria Vaccine	2,509,308,757	632,345,806,764	14,371,495,608
PRJNA679264	201	SARS-CoV-2	14,028,038,243	2,833,663,725,086	14,097,829,478
PRJNA703029	70	Trisomy 21	3,206,115,333	961,834,599,900	13,740,494,284
PRJNA600846	101	Crohn’s disease	5,928,019,591	1,197,459,957,382	11,856,039,182
PRJNA728070	10	Spinal Muscular Atrophy	743,666,190	110,857,361,100	11,085,736,110
PRJNA430406	37	Cross Tissue	2,013,009,263	402,601,852,600	10,881,131,151
PRJNA476781	468	lupus	25,280,746,391	5,056,149,278,200	10,803,737,774
PRJNA437114	43	tuberculous meningitis	2,343,938,349	463,538,572,586	10,779,966,804
PRJNA579053	8	luminal-B breast cancer	270,853,633	81,256,089,900	10,157,011,238
PRJNA664368	12	tuberculous	572,922,378	115,730,320,356	9,644,193,363
PRJNA693881	47	Viral Bronchiolitis	4,320,326,973	440,673,351,246	9,376,028,750
PRJNA329148	26	idiopathic pneumonia	1,596,881,008	239,532,151,200	9,212,775,046
PRJNA251404	16	coronary artery calcification	961,484,870	146,145,700,240	9,134,106,265
PRJNA390289	172	Chikungunya infection	5,324,807,224	1,570,379,541,476	9,130,113,613
PRJNA634938	76	SARS-CoV-2	3,422,333,457	677,622,024,486	8,916,079,270
PRJNA562305	49	cystic fibrosis	2,798,471,909	419,770,786,350	8,566,750,742
PRJNA494155	51	cervical lesions	1,450,406,148	435,121,844,400	8,531,800,871
PRJNA632871	18	globin gene depletion	759,197,460	153,357,886,920	8,519,882,607
PRJEB14743	8	leishmaniasis	277,784,189	67,426,701,419	8,428,337,677
PRJEB23048	17	visceral leishmaniasis	588,699,414	142,931,836,858	8,407,755,109
PRJNA639278	36	ADNP syndrome	987,218,170	296,165,451,000	8,226,818,083
PRJNA306908	12	Pediatric Tumors	656,614,836	98,641,641,250	8,220,136,771
PRJNA357628	13	autoimmune disease genetics	519,146,305	104,867,553,610	8,066,734,893
PRJNA591657	15	Plasmodium falciparum	783,112,666	117,466,899,900	7,831,126,660
PRJNA638653	48	Tuberculosis	1,854,820,555	370,964,111,000	7,728,418,979
PRJEB20731	13	DNASE2 mutations	599,733,466	99,295,863,162	7,638,143,320
PRJNA277352	14	multiple sclerosis	512,318,064	103,488,248,928	7,392,017,781
PRJNA511891	25	Acne	601,115,130	180,334,539,000	7,213,381,560
PRJNA504827	31	Tuberculous Meningitis	1,098,311,879	221,858,999,558	7,156,741,921
PRJNA526839	52	influenza vaccine	1,859,507,256	371,901,451,200	7,151,950,985
PRJNA680771	25	tuberculosis	621,704,238	178,360,488,275	7,134,419,531
PRJEB33892	53	Plasmodium falciparum malaria	1,875,460,009	370,990,061,196	6,999,812,475
PRJEB36928	19	Leishmania	605,785,718	132,240,365,548	6,960,019,239
PRJNA378794	38	preterm labor	1,048,641,007	264,257,533,764	6,954,145,625
PRJNA427575	2	Cryptococcal osteomyelitis	45,492,469	13,647,740,700	6,823,870,350
PRJNA454694	64	pregnancies	2,226,299,528	428,877,904,875	6,701,217,264
PRJNA384259	50	leishmaniasis	1,418,303,493	330,043,893,650	6,600,877,873
PRJEB44660	19	decompression sickness	1,218,267,812	121,826,781,200	6,411,935,853
PRJNA552286	16	high altitude	340,942,208	102,282,662,400	6,392,666,400
PRJNA305001	18	Acute mountain sickness	637,689,123	114,784,042,140	6,376,891,230
PRJNA601661	38	Cardiopulmonary bypass	1,153,286,556	228,604,842,466	6,015,916,907
PRJNA630674	79	acute respiratory illness	4,649,964,787	473,952,469,749	5,999,398,351
PRJEB41073	41	interferonopathy	1,481,311,728	245,636,835,758	5,991,142,336
PRJNA327986	36	Mycobacterium tuberculosis	1,091,879,049	205,237,031,937	5,701,028,665
PRJNA608900	8	nephrotic syndrome	515,074,459	43,781,288,465	5,472,661,058
PRJNA369684	434	Mycobacterium tuberculosis	24,108,707,793	2,362,653,363,714	5,443,901,760
PRJEB27958	28	Aicardi-Goutières syndrome	941,110,802	148,090,827,683	5,288,958,132
PRJNA397222	275	malaria vaccination	13,402,529,173	1,370,652,640,846	4,984,191,421
PRJNA493832	48	kidney transplantation	1,058,879,166	213,893,591,532	4,456,116,490
PRJNA352062	914	Mycobacterium tuberculosis	41,358,057,742	4,053,089,658,716	4,434,452,581
PRJEB27965	12	connective tissue diseases	347,784,291	52,511,397,108	4,375,949,759
PRJNA398240	22	Mycobacterium tuberculosis	1,070,313,577	92,046,967,622	4,183,953,074
PRJNA686397	271	leprosy	3,745,132,928	1,131,030,144,256	4,173,542,968
PRJNA201433	25	implant of left ventricular devices	486,115,991	97,223,198,200	3,888,927,928
PRJNA354367	24	rheumatoid arthritis	440,934,780	89,068,825,560	3,711,201,065
PRJNA401870	670	malaria infection	23,803,919,884	2,427,999,828,168	3,623,880,341
PRJNA315611	355	Mycobacterium tuberculosis	12,621,668,141	1,258,394,190,556	3,544,772,368
PRJNA380820	40	malaria	1,385,805,539	141,352,164,978	3,533,804,124
PRJNA494572	2	Immune Rep	17,746,758	6,958,474,116	3,479,237,058
PRJNA526259	99	Myalgic encephalomyelitis	1,635,104,044	330,291,016,888	3,336,272,898
PRJNA230906	6	H7N9 infection	104,404,498	18,945,387,393	3,157,564,566
PRJNA232593	47	globin depletion	925,123,735	138,768,560,250	2,952,522,559
PRJNA533086	1021	Zika	8,394,083,256	2,339,713,683,748	2,291,590,288
PRJNA679331	147	COVID-19	3,020,052,290	313,658,976,932	2,133,734,537
PRJNA613909	20	psoriasis	747,490,987	38,122,040,337	1,906,102,017
PRJNA607120	128	metabolic syndrome	1,211,648,818	182,913,643,573	1,429,012,840
PRJNA693202	26	Parkinson’s disease	364,029,613	37,131,020,526	1,428,116,174
PRJNA683803	211	HIV-infected patients	1,628,600,937	287,831,202,774	1,364,128,923
PRJNA454445	612	Circadian State	4,466,128,161	371,064,549,144	606,314,623
PRJNA459725	96	pregnancy	437,555,274	34,129,311,372	355,513,660

Projects are listed in order of bases per sample, with our current study the top listed.

### SARS-CoV-2 *vs.* Control Gene Differences

Beginning with an analysis of the first collection time point for each of the 51 patients (15 controls relative to 36 COVID-19 patients), there is a clear segregation of COVID-19 *vs.* control based on principle component analysis (**Figure 1A**). LIMMA-based differential analysis revealed 725 genes significantly higher, and 45 genes lower in COVID-19 patients *vs.* controls, with 34 genes found related to IFN signaling (**Figure 1B**). *IFI27* shows the most considerable fold change, while *DHCR24* and *FCER1A* are the most significant (**Figure 1C**). *IFI27* is a known biomarker of viral *vs.* bacterial infections connected to IFN signaling (29). *DHCR24* is an endoplasmic reticulum (ER) protein that facilitates the final step of cholesterol biosynthesis and is connected to ER stress in viral infections (30). *FCER1A* codes for the alpha subunit of IgE receptor associated with allergy response (31).

The 770 significant genes in COVID-19 *vs.* controls allows for an analysis of enriched biological processes (**Figure 1D**). From the 45 genes lower in COVID-19 patients, there is an enrichment in major histocompatibility complex (MHC) class II proteins through genes *HLA-DOA*, *HLA-DQA1*, *HLA-DQA2*, *HLA-DQB1*, *HLA-DRA*, and *HLA-DPB1*. Additional critically important lower genes include Wnt regulated master transcription factors *TCF7* and *LEF1* that are involved in T-regulatory cell differentiation, survival, and immunosuppression (32–34); *IL7R* connected to T-cell exhaustion and lymphopenia (35); *CCR7* implicated in homing and priming of T-cells and antibody response (36); and *CD3E* connected to combined immunodeficiency (37). Thus, many of the genes lower in COVID-19 are related to the acquired/adaptive immune response in COVID-19 patients. Among the additional genes involved in T-cell exhaustion, we do not see significant differences (**Supplemental Figure S1**), suggesting that the repression signals are not as easy to define pathway alterations as those activated genes.

### SARS-CoV-2 *vs.* Control Transcript Differences

Similar to gene analysis, focus on reads found on unique splicing sites for each gene allows the transcript changes to be defined (**Figure 3**). The Gencode 38 database contains 236,186 transcripts, where 64,640 are detectable in at least one sample greater than 1 TPM. The blood transcripts show the separation of control samples relative to COVID-19 patients that survived or passed away (**Figure 3A**). A total of 313 transcripts have a fold change of 2 or more and adjP <0.0005 (**Figure 3B**). Most of the top transcripts are within protein-coding transcripts of immune genes and show a significant difference for all COVID-19 patients, including lethal cases, relative to controls (**Figure 3C**). Using the biotype annotations for each transcript significantly altered relative to the blood-identified transcripts shows the highest enrichment in TR_V_genes (**Figure 3D**), which are the genes that code for the variable T-cell receptor suggesting a significant change to T-cell biology within COVID-19. Those genes with the most transcripts identified significantly different include the immune system genes *IFI27*, *TC2N*, *CASS4*, and *SMARCD3* (**Figure 3E**).

### Patient-Level Gene Panel Differences

Moving from single genes and transcripts to panels of genes, one can increase the confidence of activated biological pathways, primarily when focused on each patient. Twenty-three genes are significantly higher in the COVID-19 patients were also identified in a single-cell analysis of severe COVID-19 (38). Using the gene sets from enriched terms (**Figure 2D**), we can mark pathway activation in each individual, the total elevation of gene signatures (x-axis, **Figures 4A–C**) relative to the number of elevated genes (y-axis, **Figures 4A–C**). Multiple enrichment terms are associated with viral response, including 48 genes that have previously been connected to severe pediatric influenza infection (39). The three patients with the highest activation (patients 1, 8, 41) had lethality (**Figure 4A**). In comparison, three patients (11, 17, 40) had high activation of only a subset of influenza-connected genes *PRTN3, LTF, PGLYRP1, DEFA1B, DEFA1, DEFA4, ELANE*, and *MPO* (**Figure 4A**). This suggests more than one mechanism in activated antiviral defense, which requires future segregation analysis with a larger cohort.

Multiple enriched terms of genes higher in COVID-19 samples are connected to neutrophil processes, including secretory granules, neutrophil activation, and neutrophil extracellular traps (NETs). The 35 genes connected to NET biology associate with long-term risk of autoimmune diseases and coagulation (41, 42). NET activation’s hallmark is the upregulation of histone genes, with 44/725 (6%) of the significantly upregulated genes histones. Many of these histone transcripts are not polyadenylated (43), making their detection benefited from the total RNAseq strategy used. A total of 106 genes upregulated in COVID-19 patients are connected to secretory granules, where several of the deceased patients (51, 8, 1, and 41) had high activation (**Figure 4B**). Genes including *CEACAM6, RETN, MPO, LTF, MMP8, CEACAM8, DEFA4, OLR1, DEFA3, DEFA1B, DEFA1, ELANE* overlap secretory granule and whole-blood based severity expression in respiratory syncytial virus (44).

The highest activation of mitotic cell cycle genes was seen in patients who survived, with lower levels in some deceased patients (**Figure 4C**). Segregation of activated genes from Type I *vs.* Type II IFN response (45) suggests several deceased COVID-19 patients have elevation (**Figure 4D**). In contrast, most COVID-19 patients showed a decreased overall IFN activation signal relative to controls (**Figure 4D**). A comparison between hospitalized COVID and COVID/lethal revealed no significant genes, suggesting that lethality is either outside blood measurements, diverse, or is highly stratified with some survivors activated similar to deceased patients. The SAPSII score is a standard ICU metric that integrates multiple clinical annotations to predict disease severity and mortality risk (46). There is a significance of 1.44E-5 between COVID and COVID/Lethal at collection time point 1 for the SAPSII. A total of 17 genes (*STAT2, SPATS2L, PARP14, OLFM5P, NAPA, DDX60, CTSL, IFI44, LAMP3, APOBEC3A, UBQLNL, FANCA, HESX1, CCNA1, IL15RA, SERINC2, LAP3*) have 0.5 to 0.4 correlation to SAPSII, where the elevated expression of these genes accounts for 7 (1, 5, 8, 36, 37, 39, 43) of the patients with elevated SAPSII (**Figure 4E**). These genes are significantly associated with autoimmune phenotype genes (47, 48) (FDR >1e-5). Two of the patients (18 and 17) with high SAPSII have enrichment of a different set of 17 genes (*DEFA8P, CA1, DEFT1P, ELANE, PRTN3, AZU1, DEFA3, AHSP, ADAMTS2, DEFA4, HBD, DEFA1, CTSG, SMOX, EMID1, DAAM2, RNASE3*), associated with innate overactivation (49) (FDR 1e-10). The intricate pattern of genes relative to lethality or SAPSII score suggests a need for further segregation mechanisms more focused on individual patient response. In total, based on the 770 COVID-19 significant genes, there is a clustering of two groups of COVID-19 patients (**Figure 4F**).

From our 36 enrolled COVID-19 patients, we collected a second PAXgene tube following treatment for 12 receiving Tocilizumab, three with convalescent serum, two with Decadron (dexamethasone), five with Tocilizumab/convalescent serum, and one with Tocilizumab/Decadron. Focusing on the 770 COVID-19 significant genes, we assessed how these treatments impacted gene signatures by changes to the fold change significance, p-value significance, or the total fold change of all significant genes. Tocilizumab (12 patients) had slight alteration of elevated genes (**Supplemental Figure S2A**), while it did change several of the suppressed genes (**Supplemental Figure S2B**). Both convalescent serum and Decadron reduced the change to both elevated and suppressed genes closer to control levels. Of note, the type I/II IFN response genes show significant activation with convalescent serum treatment but not Decadron (**Supplemental Figure S2C**). Three of the five patients with before and after transcriptomes for convalescent serum treatment show initial repression of type I/II IFN response genes that are elevated following treatment (**Supplemental Figure S2D**). One of the samples that did not respond in this way was a deceased case of COVID-19. This dataset was generated as a preliminary insight into how treatments changed these COVID-19 blood signatures, and numerous future analyses from the deposited **Supplemental Data** can be performed.

### Cell Level Damage Biomarkers

While traditional RNAseq analysis tools focus on comparisons between two groups of samples, we utilize bioinformatics tools to identify unique insights for each sample that may contribute to severity (15). We began assessing genes expressed primarily within specific cell types that are significant in **Figure 1** or those with a maximum expression in the dataset using various thresholds (**Figure 5A**). As expected, many of the genes found significantly elevated within the cohort come from immune or blood-based cell types. More revealing is the elevation of genes in the blood from cells like type 2 alveolar cells, cardiomyocytes, and ciliated cells in some of the patients. These types of RNA from peripheral tissue likely are found in the blood due to release in exosomes, detachment/circulation of cells, or RNA escape during cell death. Samples from two patients who died from COVID-19 (1 and 8) have a spike in type 2 alveolar cell genes in the blood. The control sample 25 shows a spike in cardiomyocyte genes, correlating to a history of cardiovascular disease. Expanding on the immune cell types, CIBERSORTx analysis revealed segregation of lymphocytes vs. neutrophil and CD4 memory T-cell genes in COVID and COVID/lethal relative to controls (**Figure 5B**).

### Secondary Infections

We analyzed nonhuman RNA presence within each sample using KRAKEN2 tools and a database of thousands of transcriptomes from bacteria/plants/viruses/fungi. Three patients (13, 28, 36) have higher values of mapped bacterial RNA, four (12, 14, 18, 25, 28) with higher plant RNA, and two (27 and 25) with high viral RNA (**Figure 6A**). Multiple patients carried elevation of bacterial species such as *Photobacterium profundum*, *Bacillus megaterium*, and *Cryobacterium arcticum.* Bacteria like *Pseudomonas species* and *E. coli* are elevated in specific patients (patients 13/36/42, cyan, **Figure 6B**). The viruses mapped at high levels in one or more samples are human pathogenic viruses like Epstein Barr Virus (EBV. patient 45), Hepatitis C (HepC, patient 27), or markers of immune-compromised patients like Torque teno virus and Shingles seen in patient 47 (red, **Figure 6B**). We do note the detection of specific RNA reads to SARS-CoV-2 only in COVID-19 positive patients and not within controls, with the highest levels detected in the blood of patients 42, 4, and 36 (**Figure 6C**). The detection of SARS-CoV-2 using RNAseq requires the high-density performed within this study, such that for patient 42 with the highest mapping total, a total of ~10 million reads are required to observe a single read of SARS-CoV-2. The foreign RNA mapping data suggests secondary infections can contribute significantly to patient phenotypes.

### Immune Repertoire

The immune repertoire, mapped with the MiXCR tools, assesses the clonal expansion of the B and T cell variable region recombination. One can observe what pathology activates specific antibodies and T-cell receptor sequences by observing the RNA of the variable regions. The immune repertoire showed a significant elevation of reads mapped to the CDR3 region, reads per clonotype, and the max read percent in one clonotype for COVID and COVID/Lethal at collection one and further collections (**Figures 7A–D**). A total of 50 clonotypes are enriched in COVID-19 patients (cyan, **Figure 7E**), primarily consisting of IGL, IGH, and IGK chains (**Figure 7F**) that are enriched for an N-terminal set of amino acids for the IGH chain (**Figure 7G**). The COVID-19 deceased patients have an elevation of IGL clonotypes with a serine-dense sequence (orange, **Figures 7E–G**). In contrast, the COVID-19 patients who survived relative to both controls and COVID deceased patients had 38 clonotypes elevated for a conserved motif in IGK chains (red, **Figures 7E–G**).

### Patient-Level Differences

Unique biology within each patient was identified for genes or transcripts elevated four or more standard deviations from the cohort (**Figure 8**). For each sample, genes were assessed for overlapping biological function using STRING-based enrichment tools. Thus, we take the genes or transcripts significantly higher in a patient relative to the cohort and address overlapping biological function from those genes using GO enrichment statistics, representing a common statistical strategy applied within precision medicine. This yields insight for each sample from overactive components of the immune system, potential neurological complications, and cardiovascular impairments (**Figure 8**). This precision insight into COVID-19 patients and controls suggests an underappreciated role of cell type damage resulting in elevation of RNA into the blood, secondary infections including other viruses, and unique biological responses involved in outcomes of infections. This can be highlighted in patient 1, who had lethal COVID-19. In this patient, we observe high mapping for organ severity SAPSII, significant genes including those involved in overlap to severe influenza or neutrophil biology, neutrophil to lymphocyte ratio, genes connected to blood group antigens/erythroblastosis, and RNA unique to Type 2 Alveolar cells. Yet this patient maintained average to below-average values for mapping secondary infections and expanding clonotypes within B and T-cells. Other deceased patients, such as patient 51, presents similar to COVID-19 survivors, with lower SAPSII, gene activation in nearly every category, and minimal reads from cell-specific RNA but show activation of secondary viral EBV and a unique over-activation of a subset of significant genes, suggesting that COVID-19 pathology is not one mechanism but rather can result in severity through different fundamental mechanisms.

### Transcriptomics Relative to COVID-19 HGI GWAS

This new high-density transcriptome of severe COVID-19 has additional utility outside of precision transcriptomics, namely in being a tool in further filtering public data. To show this potential, we have filtered the severe COVID-19 Genome-Wide Association (GWAS) data from the COVID-19 HGI (12). Using the top 1,000 variants based on the lowest p-value, SNP Nexus (28), and various genome level annotations, including GeneHancer and RegulomeDB, allowed top variants’ prioritization (**Supplemental Figure S3A**) to gene annotations. Based on Combined Annotation Dependent Depletion (CADD) score, chr19:10317045 T/A (p-value 1.52E-10) ranks the highest (CADD 17.68) with an expression quantitative trait locus (eQTL) to *KRI1*. The 3p21.31 region has been the center of many investigations since its original report as associated with COVID-19 severity (50). The most highly associated 12 SNPs (p-value <10^-50) of the region remain weakly associated to any genes, with rs71325088 (p-value 9.75E-61) scoring high in CADD (10.54) but little biological connection to COVID-19 severity. The SNP rs13082697 (p-value 2.23E-32) found in the 3p21.31 region also has a high CADD score (16.25) with some potential eQTL for the gene *CXCR6*. This suggests the need for additional tools to narrow the search of causal genes than many functional annotation tools can currently provide.

The list of 115 genes connected to the top 1,000 SNPs was assessed through our differential data of hospitalized COVID-19 blood transcriptomics *vs.* controls (**Supplemental Figure S3B**). Seven of our significant genes higher (*GALNT14, OAS1, CCRL2, OAS3, CCR1, OAS2, SIPA1L2*) in COVID-19 and two lower (*HLA-DPB1, HLA-DQA2*) were identified to overlap. The larger region of 3p21.31 with the significant association has SNP rs3181080 (p-value 7.13E-11), with a perfect RegulomeDB score, an eQTL for the significant *CCR1* gene, and GeneHancer linkage to the significant *CCRL2* gene. *CCR1* is associated with antiviral defense, and its deletion attenuates pathological overactive immune responses in the respiratory system (40, 51). SNP rs2384072 ranks as 1b in RegulomeDB, an eQTL for *OAS1* connected to viral susceptibility (52). A full data insight and gene matrix for the GWAS regions can be found in the online data (https://doi.org/10.6084/m9.figshare.13524275.v1). The data suggest the utility of our RNAseq in future filtering and understanding of immune and viral response genetics.

## Discussion

Viruses are intracellular pathogens that eradication is largely dependent on the host’s immune system (53). The host-pathogen relationship is complex, even more so in aging patients. This leads to variability in clinical outcomes in viral infections and has been particularly notable within COVID-19 patients. This work shows that clinical severity can be correlated to gene markers; however, we could not differentiate by lethality likely due to our sample size and the complicating effect of patient comorbidities and divergent responses to treatments for COVID-19 that were used based on the best evidence at that time. Despite this limitation, we were able to show some relationship to SAPSII scores which control for comorbidities.

**Figure 9:**
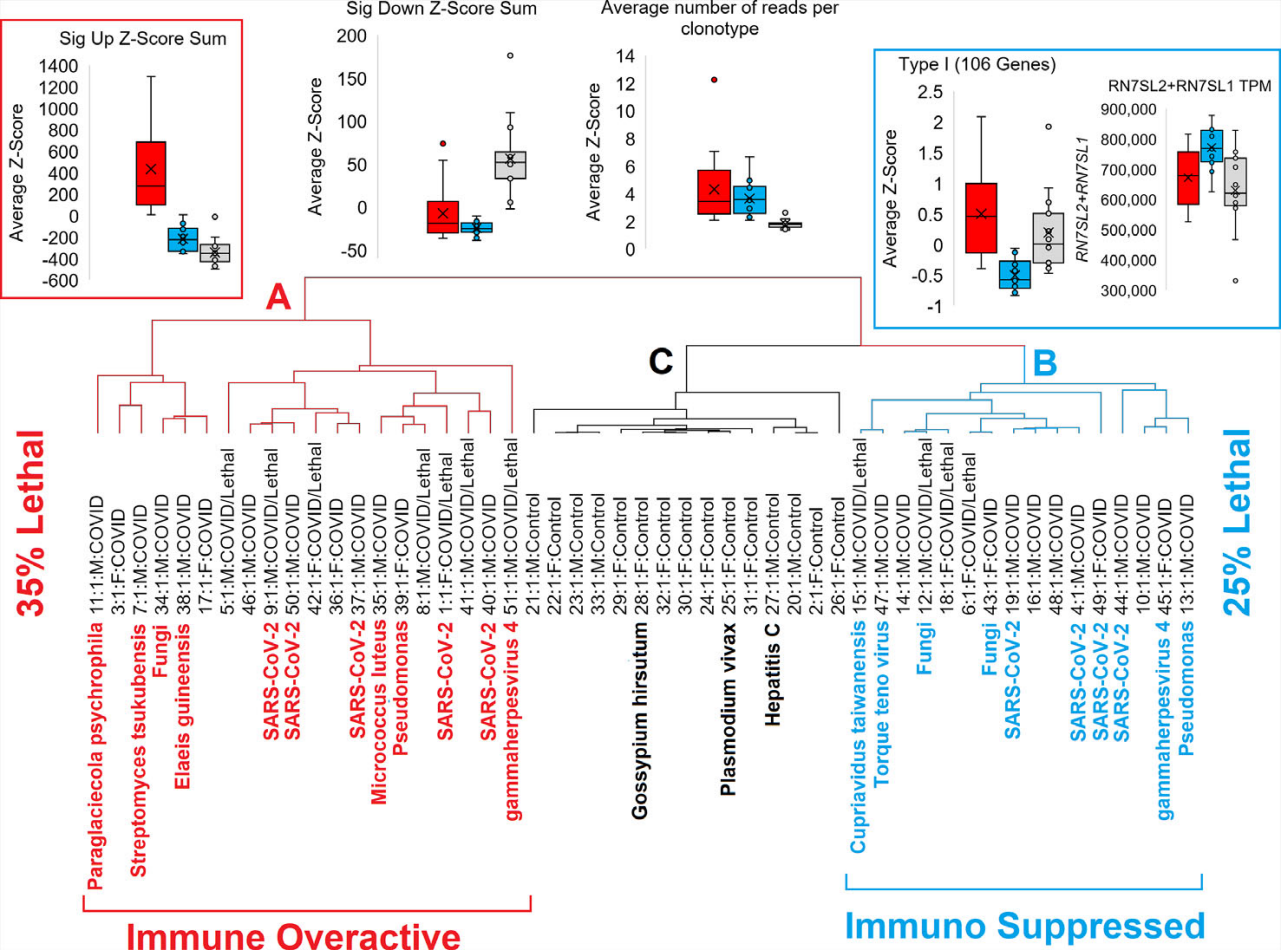
Schematic of two different groups of COVID patients relative to controls. Red represents the immune overactive COVID-19 patients (group A), cyan the immune suppressed patients (group B), and controls cluster in black (group C).

Utilizing all of the mapped data, we can cluster COVID-19 patients into two distinct groups that cluster separately from controls (**Figure 9**). All COVID-19 patients show strong downregulation of genes and clonal expansion of the acquired immune system. One group of COVID-19 patients (denoted as group A) have the highest activation of our significant gene list, suggesting an overactive immune system that might benefit the most from immune-suppressive drugs. The second group of COVID-19 patients (denoted as group B) have strong suppression of Type I IFN response and an elevation of *RN7SL2* and *RN7SL1* genes collectively known as 7SL RNA, both connected to viral packing and trafficking (54, 55) and known to interact with the SARS-CoV-2 NSP8 and NSP9 proteins to impact viral trafficking to the cell membrane (56). This group B set of patients may benefit from higher suspicion for nosocomial infections and respond better to immune-stimulating agents that activate the Type I IFN response, such as convalescent serum, as they appear to be an immune-suppressed group. This is highlighted by the patient 47 inclusion in the group that had an elevation of the *Torque teno* virus, which can be associated with immune system suppression (57). The Type I IFN response has been central to COVID-19 pathology, with numerous treatment strategies being tested (58). Consistent with our findings, protein cytokine profiles have also suggested that COVID-19 has impaired Type I IFN response (59, 60), but we suggest here that this is not common to all COVID-19 hospitalized patients. Amongst the top genes we see altered in COVID-19 is the interferon response protein *ISG15*, which is also known to be a direct target of the SARS-CoV-2 NSP3 protease for the removal of ISGylation from target proteins such as the MDA5 sensor that can regulate the intracellular IFN response by viruses (61–63).

The utility of PAXgene tube total RNA at such a high-depth sequencing provides a unique resource to both the clinical and scientific community that is currently not being utilized. This high sequencing depth allowed us to show that many hospitalized COVID-19 patients had distinct factors that were not clinically detectable, including secondary infections and peripheral organ damage. Even in our small sample size, we were able to show by serial assessment of data that treatments such as convalescent serum and dexamethasone (Decadron) dampened some of the signatures of COVID-19 hospitalized infections. While the sample size before and after therapy was limited, the signatures seen would suggest the need to perform additional analysis using similar or lower depth RNAseq. Patients were enrolled in the study before treatment, and the observational study did not influence the clinical course. Therefore, as COVID-19 treatments changed early in the pandemic, our groups of patients for each treatment were not balanced. Our in-depth ability to evaluate therapeutic responses could provide valuable information to a treating physician in a timely fashion. RNA sequencing (RNA-Seq) provides transcriptome profiling that allows an unbiased survey of the entire transcriptome in a high-throughput manner. The deeper coverage and single-nucleotide resolution of our RNAseq provides a platform to determine differential expression of genes or isoforms that could provide a deeper understanding of the host-pathogen relationship. The depth shows the uniqueness of individual patients with the same disease, a critical need in precision medicine.

Detection of RNA from other pathogenic or opportunistic viruses and bacteria (*EBV, Shingles, Pseudomonas, E. coli*) brings about an interesting question of whether COVID-19 alone drove hospitalizations or whether these other agents contributed or even resulted in the hospitalization. We know that all COVID-19 patients displayed the classical symptoms of lung infections and were hospitalized primarily for COVID-19. We, therefore, assume that these other agents contribute to the severity of phenotype but were not the leading cause of hospitalization.

Our findings overlap with other COVID-19 transcriptomic studies but suggest caution in extrapolating single group comparisons regarding either severity or lethality. Multiple factors can independently contribute to the outcome through diverse mechanisms. RNAseq in infected epithelial cells, peripheral tissues, and blood has suggested a mechanism of decreased IFN response (5), yet we show this to occur only in a subset of patients. Studies show a subset of COVID-19 patients to develop autoantibodies against type I IFN (11, 64), so further work is warranted to show if autoantibodies correlate to decreased IFN signaling. Profiles of single-cell RNAseq from patient blood samples confirm the suppression of B- and T-cells (lymphopenia), T-cell exhaustion, and TNF/IL1β–driven inflammation signature (65–68) not typical to all mortalities in this study. Targeted immune repertoire sequencing strongly supports our findings that the B-cell repertoire has clonal expansion (69), further expanding in later collection time points. To the best of our knowledge, this is the first transcriptomic study performed at this high depth of reads with the integration of broader multidimensional bioinformatic analyses focused more on the patient level insights rather than a cohort-based approach for infectious disease and immune response.

An improved understanding of severe COVID-19 disease pathogenesis and the patient’s physiologic and immunologic state could help identify patients at significant risk for complications, guiding precision therapeutic approaches. RNA transcriptomic analysis in hospitalized patients can guide clinical trials and individualized treatment with various immunomodulators for COVID-19 and future emerging infectious diseases. The value of our study is that it highlights how transcriptomics provides a detailed snapshot of the patient’s entire immunologic state that could have value in immediate clinical decision-making. In the long term, this data may also help us understand and more effectively predict, prevent, and treat long-term sequelae of this and other viral infections. Thus, additional studies of high-density precision transcriptomics in patients with COVID-19 infection are greatly needed. 

## Data Availability Statement

The datasets presented in this study can be found in online repositories. The names of the repository/repositories and accession number(s) can be found in the article/**Supplementary Material**.

## Ethics Statement

The studies involving human participants were reviewed and approved by Spectrum Health IRB. The patients/participants provided their written informed consent to participate in this study.

## Author Contributions

Designed and oversaw experiments: JP, NH, DC, CB, and SR. Performed RNAseq: RK, NA, BF, LT, and TM. Analyzed transcriptomic data: JP, WF, AF, JB, NK, AD, RP, NH, JM, AE, and RS. Processed patient data: NH, CL, ML, EV, SP, CB, and SR. Contributed to interpretation of data: BE, AH, BC, JC, DS, and RO. Generated figures and wrote the manuscript: JP, NH, and SR. All authors contributed to the article and approved the submitted version.

## Funding

This work was supported by the Spectrum Health and MSU Alliance Corporation, Spectrum Health Foundation, National Institutes of Health (K01ES025435 to JP, R01GM108618 to JC), and Michigan State University.

## Conflict of Interest

JP performed a summer sabbatical in 2020 for AbbVie Inc, receiving hourly pay. RK, NA, BF, and LT were employees of Ambry Genetics during this study. NK is an advisory board member for Horizon pharmaceuticals and Pharming Healthcare. None of these listed conflicts were directly related to the current work.

The authors declare that the research was conducted in the absence of any commercial or financial relationships that could be construed as a potential conflict of interest.
