# Supplementary material for: High-Density Blood Transcriptomics Reveals Precision Immune Signatures of SARS-CoV-2 Infection in Hospitalized Individuals
Source: Front Immunol. 2021 Jul 16;12:694243. doi: 10.3389/fimmu.2021.694243 (PMC8322982; doi:10.3389/fimmu.2021.694243)
Supplement: Supplementary file 1 [file DataSheet_1.pdf]

Supplemental Figures

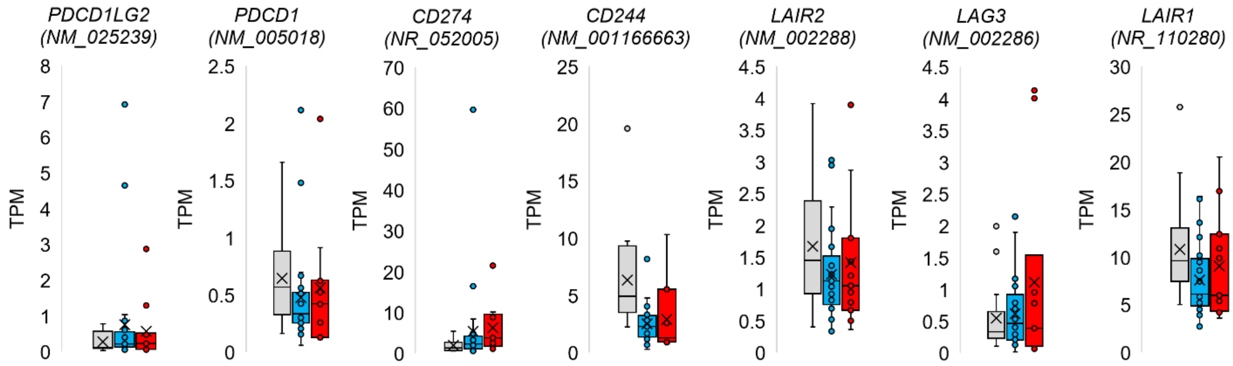

Figure S1 Exhaustion marker gene expression.

Box and whisker plots for control (gray), COVID-19 (cyan), and COVID-19/lethal (red) expression for seven different markers of T-cell exhaustion.

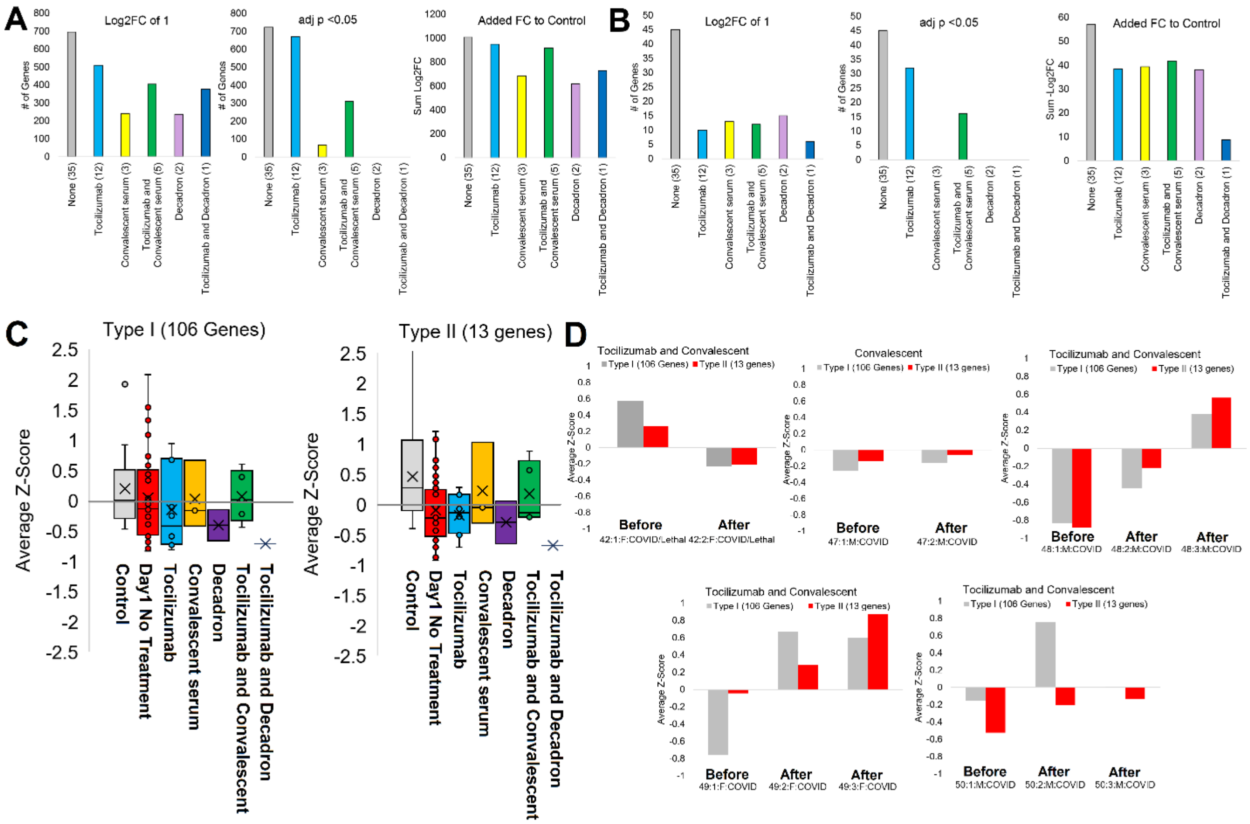

Figure S2 Response in patients to various therapies.

A-B) The change in significant up (K) or down (L) regulated genes for samples treated with nothing (gray), Tocilizumab (cyan), convalescent serum (yellow), Tocilizumab and convalescent serum (green), Decadron (purple), or Decadron and convalescent serum (blue). The left panel shows the number of genes in each group that reach significant fold change. The middle panel shows the number of genes in each group that reach a significant adjusted p-value, calculated based on Limma differential analysis. The right panel shows the added fold change value for all significant genes.

C) The break down of Type I IFN (left) and Type II IFN response (right) genes based on the average z-score for each of the genes in the groups. Breakdown is based on drug treatments relative to controls.

D) Before and after treatment with convalescence serum for the average Z-score for Type I IFN (gray) and Type II IFN response (red) genes in five different patients.

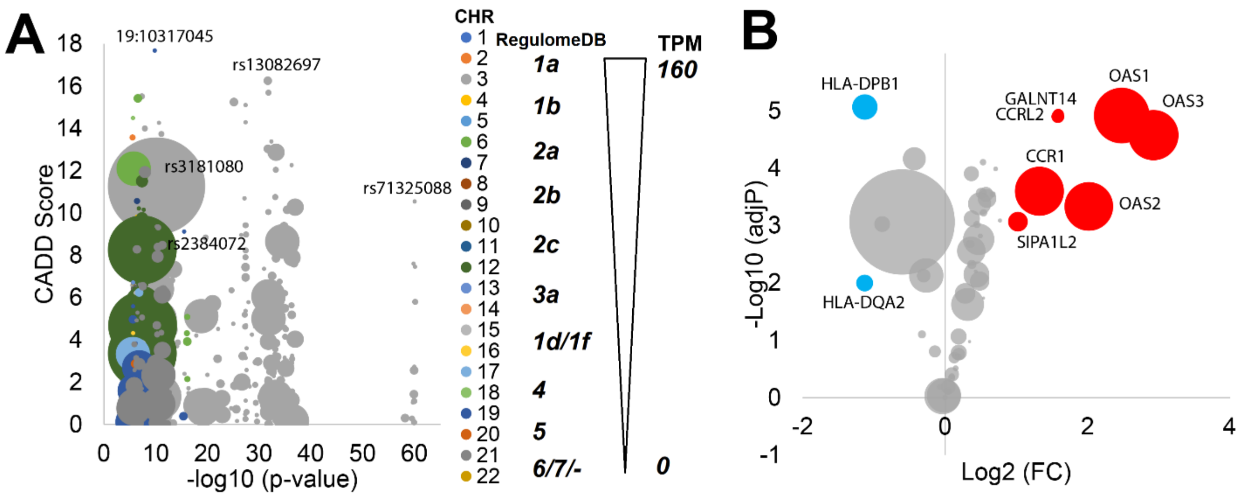

Figure S3 Gene signatures for COVID-19 assessed relative to known COVID-19 GWAS.

A) Assessment of variants from the severe COVID-19 HG GWAS showing their significant p-value (x-axis) relative to CADD (y-axis) or RegulomeDB score (radius of spot).

B) The expression of genes linked to GWAS loci in panel G showing the log2 fold change (x-axis), adjusted p-value (y-axis), and the average TPM of samples (radius of spot). Genes that fall in the significant group (Figure 1B) are colored red (higher) or cyan (lower).

Supplemental Excel file can be found at https://doi.org/10.6084/m9.figshare.13524275

Description of Supplemental Excel File Tabs

Samples: A full breakdown of information for each of the samples with RNAseq in the current manuscript. Combined name= list of patient:collection:sex:group (this name is used in labeling of figures and tables for each patient); Patient= patient numbers 1-51; Collection= the number tube from each patient; Sex= male (M) or female (F); Group= Control, COVID, or COVID/Lethal; Age Range= Binned groups for the age of patient; Race= the race of the sample; SAP= combined annotation of pathology; Treatment before sample= any treatment recorded before the sample collection; Yield(Mbases)= the total number of sequenced bases in each sample; Clusters: the number of unique cluster spots sequenced in each sample; % perfect barcode= the percent of reads in sample where the barcode matches expected; One mismatch barcode= percent of reads in sample with a single mismatch in the barcode; %>-Q30 bases= percent of reads with a quality score greater than or equal to 30; Mean Quality Score= the average quality score for reads of each sample; TPM Sum (for multiple gene sets): added TPM counts for genes within each gene set; Z-Score Sum= added z-score levels for gene sets; Neutrophils x T cells CD4 memory activated= the CIBERSORTx absolute values for each multipled together for each sample; T cells CD4 memory resting + T cells CD8 + B cells memory= the CIBERSORTx absolute values for each added together; KRAKEN2 Viridiplantae= normalized counts of total plant mapped reads in each sample; KRAKEN2 Bacteria= normalized counts of total bacteria mapped reads in each sample; KRAKEN2 Virus= normalized counts of total viral mapped reads in each sample; KRAKEN2 Fungi= normalized counts of total fungal mapped reads in each sample; KRAKEN2 SARS-CoV-2= normalized counts of mapped reads in each sample for SARS-CoV-2; KRAKEN2 Outlier= Species that are found enriched within each sample; Number of Genes Z-score >4 Collection 1= genes in each sample with a z-score greater than 4; Gene GO Terms (FDR <.1) Collection 1= terms enriched in z-score genes >4 for each sample; Number of Transcripts Z-score >4= transcripts in each sample with a z-score greater than 4; Enriched Biotypes >2= Gencode based biotypes that are enriched with in patients transcripts >4; Cell Type Specific Gene Enrichment= cell types that have gene expression increased within each patient; % of reads aligned MiXCR= the CDR3 reads identified out of all RNAseq reads of each sample; Mean V extension length= the mapped length of V region for each sample; Mean J extension length= the mapped length of J region for each sample; Final clonotype count= the total number of distict clone types identified from CDR3 of each sample; Average number of reads per clonotype= the number of clones within each clonotype averaged for each sample; clonotypes with >0.1= clonotypes that contain 10% or more of all the mapped CDR3 reads; clonotypes with >0.01= clonotypes that contain 1% or more of all the mapped CDR3 reads ; clonotypes with >0.001= clonotypes that contain 0.1% or more of all the mapped CDR3 reads; clonotypes with >0.0001= clonotypes that contain 0.01% or more of all the mapped CDR3 reads; Max reads per clonotype= the higher percent of reads mapped to any clonotype found within a sample.

Demographics (pre-existing): Analysis of medical records revealed the following pre-existing conditions for COVID-19 patients (n=38). Patients may have more than one condition listed; a) Includes Coronary Artery Disease, atrial fibrillation, Congestive heart failure (CHF), Hx of aortic valve endocarditis, Myocardial infarct (HCC), Bradycardia & Cardiomyopathy. b) Includes COPD, Upper Respiratory Infection, Pulmonary embolism, PPD positive (Tuberculosis), Bronchitis, Pneumonia, Pulmonary hypertension, & Sarcoidosis. c) Includes Breast cancer,

Prostate cancer, Skin cancer & Bone cancer. d) Includes Hyperlipidemia, Hypercholesteremia, & Dyslipidemia.

Demographics (admissions): Analysis of medical records revealed the following admissions diagnoses for COVID-19 patients (n=38). * Patients may have more than one condition listed; a) Includes suspected COVID-19; b) Non-ST-elevation myocardial infarction.

Gene Mapping: The transcript per million (TPM) mapping data for each sample from Salmon quasi-alignment. Each sample has values listed for every gene. Both the accession and gene names are highlighted in gray.

Sig Up: The genes from Figure 1A in red that are significantly higher in COVID-19 patients vs controls. Symbols= Gene name; logFC= the log2 fold change between COVID-19 and control patients; AveExpr= the average expression of gene; P.Value= p-value between COVID-19 and controls; adj.P.Val= adjusted P-value between COVID-19 and controls. B= B factor; Name= long name of the symbol.

Enriched-Up: The Sig Up gene list for gene ontology enriched terms generated from STRING analysis. Enriched Group: the classification group from STRING for the term; term ID= a searchable code for the enrichment term; term description= the term information; observed gene count= the number of genes from Sig Up tab that is found listed for the term; background gene count= number of genes within the genome that are listed for the term; strength= STRING based ranking of terms; false discovery rate= the significance of the enrichment; -log10= the negative log 10 of the FDR; matching proteins in your network= protein accession codes for genes linked to term; matching proteins in your network= the genes found matching between our significant genes and the term gene list.

Sig Down: The genes from Figure 1A in blue are significantly lower in COVID-19 patients vs. controls. Columns are the same as in the Sig Up tab.

Enriched-Down: The Sig Down gene list for gene ontology enriched terms generated from STRING analysis. Columns are the same as in the Enriched-Up tab.

Top Term Groups: Those enrichment terms from Figure 1D.

Cell Type Genes: Genes from the Human Protein Atlas Single Cell Type Atlas (www.proteinatlas.org/humanproteome/celltype). Accession number and gene name are highlighted in gray. The cell type associated with the gene is in red. In yellow are the genes that are within the significant genes for COVID-19 vs. control patients. In cyan are statistics from our patient cohort, including the maximum value (Max), average, standard deviation (StDev), and the z-score of the maximum value (Max Z).

Cell Type Summed: The summed gene-level TPM or Z-score (Z) for each cell type. In gray is each sample identifier. Column 1 lists the cell type, column 2 lists whether the column values are for summed TPM or Z-score (Z), and column 3 lists how many genes there are for each cell type.

CIBERSORTx: The absolute prediction from CIBERSORT tools for each of 22 immune cell types based on our gene expression matrix. In gray are the name of each sample and CIBERSORTx statistics of each sample. Following are the values for each immune cell type.

Gencode v35: The transcript per million (TPM) mapping data for each sample from Salmon quasi-alignment to the Gencode version 35 database. In gray are details of each transcript, including the

transcript identifying (Transcript Accession), gene accession, the Ensembl based transcript and gene information, the length of the transcript, and the annotated biotype.

Kraken2: Normalized mapping data for read alignment to each level of annotation of evolution. Normalization was performed to transcripts mapped per million human aligned reads (red). In gray are details of each transcriptome annotation, including the identity (name of level), the phylogenetic code, and the phylogeny level. In yellow is the number of samples that have values greater than 0.

Treatment to Control: All genes analyzed by Network analysis showing the change of gene expression within each group relative to controls. The first two columns show the symbol and EntrezID for the gene. In yellow is if the gene is within our significant gene list of COVID-19 vs. controls. Rotating between gray and no highlights are the changes for various treatment groups starting with no treatment (gray), Tocilizumab (no highlight), Convalescent serum (gray), Tocilizumab and Convalescent serum (no highlight), COVID-19 w/ Decadron (gray), Tocilizumab and Decadron (no highlight).

MiXCR_stats: Output values for various MiXCR statistics for each sample.

MiXCR_clonotype: The Clonotypes identified in cyan, red, or orange in Figure 2G. Each clonotype lists stats in yellow, including NA Hit= the nucleic acid sequence, AA Hit= corresponding amino acid sequence, Len= length of insert, Max % Clonotype= the highest percent of reads from samples, Samples >0.001, the number of samples with more than 0.1% of total CDR3 reads that match to clonotype, MaxCOVID-MaxControl= the maximum value from COVID samples (including COVIDLethal)minus the maximum value of controls, Log2(COVID/Control)= the log2 fold change for the average of COVID or Control samples; MaxLethal-MaxNonLethal= the maximum value of COVIDLethal samples minus the maximum values of COVID nonLethal samples, Log2(Lethal/NonLethal)= the log2 fold change of average values for COVIDLethal over COVID nonLethal. Following stats are the percent of reads matched to each clonotype for every sample with controls highlighted in gray, COVID collection 1 in white, COVID collection 2 or more in blue, COVIDLethal collection 1 in red, and COVIDLethal collection 2 or more in orange.

Outlier Genes >4 Collection 1: The genes found in each sample that have a z-score greater than 4.

Outlier Transcripts: The transcripts found in each sample that have a z-score greater than 4.

COVID GWAS: A breakdown of the top 1,000 A2_ALL COVID-19 Host Genetics Initiative (October 2020 release) genome wide association variants. #CHR= the chromosome of variant; POS= position on CHR based on hg38; rsid= the accession code for the variant if one has been given to the variant; Distance to closest Snp= the basepair distance to the closest SNP before this one; REF= reference basepair of the genome at position; ALT= the alternate basepair found for variant; all_inv_var_meta_p= the p-value from the COVID-19 HGI, which is highlighted in yellow; 1KGen (gray highlight)= the allele frequency of various populations from the 1,000 genomes project (SAS= South Asian, EAS= East Asian, EUR= European, AMR= American, AFR= African); gnomAD Genomes= Allele frequencies from the gnomAD genomes population (FIN= Finnish European, AMR= American, OTH= Other, AFR= African, EAS= East Asian, NFE= Non-Finnish European); Near Genes= the closest annotated gene to the variant as annotated by SNPnexus; GWAS Hits= number of GWAS traits associated with this variant as annotated by SNPnexus; GWAS= the traits associated with this variant as annotated by SNPnexus; CADD=

Combined Annotation Dependent Depletion (CADD) function score (https://cadd.gs.washington.edu/); ClinVar= If a variant is within the ClinVar database it lists the predicted functional annotation; TARBASE= functional annotation of microRNA; FunSeq2= annotation of noncoding variant potential impact; PolyPhe2/SIFT= prediction if a coding variant impact function; ensembl (gray)= the details of location of variant based on ensembl annotations; CpG= if the variant is located within a CpG rich region that might have gene regulation potential; GeneHancer (gray)= extracted data from the human genome browser for GeneHancer details; ENCODE= details of overlapping ENCODE datasets for each variants; ROADMAP (gray)= details of overlapping roadmap epigenomics datasets for each variants; RegulomeDB Score= score from regulomedb.org for each variant (1a=eQTL + TF binding + matched TF motif + matched DNase Footprint + DNase peak, 1b=eQTL + TF binding + any motif + DNase Footprint + DNase peak, 1c=eQTL + TF binding + matched TF motif + DNase peak, 1d= eQTL + TF binding + any motif + DNase peak, 1e= eQTL + TF binding + matched TF motif; 1f= eQTL + TF binding / DNase peak, 2a= TF binding + matched TF motif + matched DNase Footprint + DNase peak, 2b= TF binding + any motif + DNase Footprint + DNase peak, 2c= TF binding + matched TF motif + DNase peak, 3a= TF binding + any motif + DNase peak, 3b= TF binding + matched TF motif, 4 TF= binding + DNase peak, 5= TF binding or DNase peak, 6= Motif hit, 7= Other).

Genes Linked to GWAS: Expression of genes linked to data within the COVID GWAS tab. Highlighted in yellow is if genes were within our significant gene list of COVID-19 vs. controls. The average TPM all shows the average value for each gene over all of the samples. Log2FC and adj.P.Val show the differences between COVID-19 vs. controls at collection time point 1.
